# Genomic Sequence of a Ranavirus Isolated from Short-Finned Eel (*Anguilla australis*)

**DOI:** 10.1128/genomeA.00843-16

**Published:** 2016-08-18

**Authors:** Kuttichantran Subramaniam, Anna Toffan, Elisabetta Cappellozza, Natalie K. Steckler, Niels J. Olesen, Ellen Ariel, Thomas B. Waltzek

**Affiliations:** a Department of Infectious Diseases and Pathology, College of Veterinary Medicine, University of Florida, Gainesville, Florida, USA; b National Reference Laboratory for Fish Diseases, Istituto Zooprofilattico Sperimentale delle Venezie, Legnaro, Italy; c National Veterinary Institute, Technical University of Denmark, Frederiksberg C, Denmark; d College of Public Health, Medical and Veterinary Sciences, James Cook University, Townsville, Queensland, Australia

## Abstract

The short-finned eel ranavirus (SERV) was isolated from short-finned eel imported to Italy from New Zealand. Phylogenomic analyses revealed that SERV is a unique member of the genus *Ranavirus*, family *Iridoviridae*, branching at the base of the tree near other fish ranaviruses.

## GENOME ANNOUNCEMENT

Short-finned eel ranavirus (SERV) was isolated in 1999 from short-finned eel (*Anguilla australis*) imported to Italy from New Zealand (1). Ten apparently healthy eels weighing between 200 and 300 g were processed for diagnostic virology as part of a routine screening of imported live fish. Internal organ homogenates seeded onto epithelioma papulosum cyprini (EPC) cells resulted in CPE characterized by focal plaques. Negative stain electron microscopy performed on infected culture supernatant revealed iridovirus-like particles. Infected cell cultures reacted positively in a direct immunofluorescence assay using a rabbit polyclonal serum against the fish ranavirus, *European catfish virus* (ECV, [2]), suggesting that the replicating agent was a member of the genus *Ranavirus*.

A sixth passage of the SERV isolate was amplified in EPC cells maintained in modified Eagle’s medium with 10% fetal bovine serum at 23°C. Inoculation of EPC cells at high multiplicity of infection provided material from an eighth passage harvested after 96 h. Cell culture supernatant was clarified at 3,000 × *g* for 20 min, and total nucleic acids were purified using a DNeasy blood and tissue kit (Qiagen). A DNA library was prepared using a Nextera XT DNA kit (Illumina) and sequenced using a V3 chemistry 600-cycle kit on a MiSeq platform (Illumina). *De novo* assembly of 10,752,536 paired-end reads in SPAdes (3) produced a contiguous consensus sequence of 126,965 bp with a G+C content of 55.64%. The quality of the genome assembly was assessed by mapping the reads back to the consensus sequence in Bowtie 2 (4) and visually inspecting the alignment in Tablet (5). A total of 6,417,927 reads (59.69%) aligned at an average coverage of 10,720 reads per nucleotide.

The genome of SERV was annotated using GATU (6) with *Frog virus 3* (GenBank accession no. NC_005946) as the reference. Additional putative open reading frames (ORFs) were identified using GenemarkS (7). A total of 111 putative ORFs were predicted in SERV compared to other related fish ranaviruses, including 100 in *Epizootic hematopoietic necrosis virus* (EHNV, GenBank accession no. NC_028461), 135 in ECV (GenBank accession no. KT989884), and 136 in European sheatfish virus (ESV, GenBank accession no. JQ724856). An analysis of locally collinear blocks in Mauve (8) revealed that the genomes of SERV, EHNV, ECV, and ESV are collinear. Phylogenetic analyses based on the concatenated nucleotide sequences of the 26 *Iridoviridae* core genes (9) revealed that SERV forms a distinct branch at the base of the ranavirus tree near other fish ranaviruses (e.g., EHNV, ESV, and ECV) with only the highly divergent Santee-Cooper *ranavirus* and grouper iridoviruses splitting off earlier.

Although some fish ranaviruses can cause considerable mortality in aquaculture (e.g., EHNV, ECV, and grouper iridoviruses), others like SERV are rarely detected and thus their impacts are unknown (10). Bath challenges with SERV resulted in significant mortality in northern pike *Esox lucius* fry (11) versus no appreciable disease in juvenile black bullheads *Ameiurus melas* (12). Thus, SERV appears to have minimal impacts on the health of some hosts, including short-finned eel, while causing disease in others.

### Accession number(s).

The complete genome sequence of SERV has been deposited in GenBank under accession number KX353311.
